# Development and Calibration of Pressure-Temperature-Humidity (PTH) Probes for Distributed Atmospheric Monitoring Using Unmanned Aircraft Systems

**DOI:** 10.3390/s22093261

**Published:** 2022-04-24

**Authors:** Karla S. Ladino, Michael P. Sama, Victoria L. Stanton

**Affiliations:** 1 Department of Biosystems and Agricultural Engineering, University of Kentucky, Lexington, KY 40546, USA; karla.ladino@uky.edu; 2 Applied Statistics Laboratory, University of Kentucky, Lexington, KY 40546, USA; victoria.stanton@uky.edu

**Keywords:** distributed atmospheric monitoring, unmanned aircraft systems, barometric pressure, temperature, relative humidity, embedded systems, calibration

## Abstract

Small unmanned aircraft systems (UAS) are increasingly being used for meteorology and atmospheric monitoring. The ease of deployment makes distributed sensing of parameters such as barometric pressure, temperature, and relative humidity in the lower atmospheric boundary layer feasible. However, constraints on payload size and weight, and to a lesser extent power, limit the types of sensors that can be deployed. The objective of this work was to develop a miniature pressure-temperature-humidity (PTH) probe for UAS integration. A set of eight PTH probes were fabricated and calibrated/validated using an environmental chamber. An automated routine was developed to facilitate calibration and validation from a large set of temperature and relative humidity setpoints. Linear regression was used to apply temperature and relative humidity calibrations. Barometric pressure was calibrated using a 1-point method consisting of an offset. The resulting PTH probes were less than 4 g in mass and consumed less than 1 mA when operated from a 5 VDC source. Measurements were transmitted as a formatted string in ASCII format at 1 Hz over a 3.3 V TTL UART. Prior to calibration, measurements between individual PTH probes were significantly different. After calibration, no significant differences in temperature measurements across all PTH probes were observed, and the level of significance between PTH probes was reduced. Actual differences between calibrated PTH probes were likely to be negligible for most UAS-based applications, regardless of significance. RMSE across all calibrated PTH probes for the pressure, temperature, and relative humidity was less than 31 Pa, 0.13 °C, and 0.8% RH, respectively. The resulting calibrated PTH probes will improve the ability to quantify small variations in ambient conditions during coordinated multi-UAS flights.

## 1. Introduction

The advancement of small unmanned aircraft systems (UAS), commonly referred to as drones, has provided new opportunities to study the lower atmospheric boundary layer. Sensors are now commonly deployed on UAS to study atmospheric properties, including temperature, pressure, humidity, wind velocity [1], and turbulence [2] at a wide range of spatial scales [3]. UAS-based in-situ measurements can also support weather simulation modeling by providing validation data at finer spatial resolutions than were previously available [4,5]. Other practical applications of UAS-based atmospheric monitoring include direct observations of volatile organic compounds and other gaseous emissions from wildfires [6,7], which would further benefit from instrumentation to quantify the flow field that transports these emissions.

Several multidisciplinary collaborations in the UAS-based atmospheric research domain have emerged in the past decade. The Collaboration Leading Operational UAS Development for Meteorology and Atmospheric Physics (CLOUD-MAP) project [8] was one of these efforts and sought to catalyze the use of drones in atmospheric research by developing systems and operational expertise [9]. A noteworthy outcome of CLOUD-MAP was the concept of a three-dimensional network of atmospheric monitoring stations deployed on autonomous UAS [10]. The operational capacity developed through CLOUD-MAP helped enabled larger and more targeted experiments to be conducted. In 2018, a collaboration between CLOUD-MAP and the International Society for Atmospheric Research Using Remotely-Piloted Aircraft (ISARRA) resulted in the Lower Atmospheric Profiling Studies at Elevation–A Remotely Piloted Aircraft Team Experiment (LAPSE-RATE) [11]. LAPSE-RATE was conducted over one week in the San Luis Valley in Colorado, USA, resulting in large publicly available datasets of coordinated ground- and UAS-based distributed atmospheric measurements [4,12,13,14,15,16,17,18,19,20].

The UAS and instrumentation used during LAPSE-RATE varied between institutions. An intercomparison experiment was conducted to account for these differences by benchmarking sensors to a reference instrument [21]. Thirty-eight UAS carrying 23 unique sensors were compared to a mobile ground station. Results showed general agreement with the reference instrument but also highlighted the need for improvements in data collection methods. Distinguishing sources of measurement error was difficult due to the wide variety of UAS platforms, sensors, and instrumentation methods. Some of the sensors used were commercially available systems designed for UAS deployment (e.g., iMet-XQ2, International Met Systems, Grand Rapids, MI, USA), while others were custom-built [22,23].

Quantifying and mitigating error through calibration is critical when collecting distributed atmospheric measurements, where the error in uncalibrated instruments may exceed the anticipated spatiotemporal variability in parameters measured. Commercial embedded sensors commonly provide an output in arbitrary units (e.g., a voltage signal or digital value) that must be converted to the sensed parameter with associated engineering units using an empirical function. This function is common across all sensors of the same model and does not consider individual sensor variability, how the sensor has been integrated into a system, or address possible measurement drift over time. The shortcomings of relying on nominal outputs are evident in [21], where large offsets in relative humidity that exceed manufacturer specified accuracy were present.

One potential solution to mitigate measurement error is standardizing the sensors used during team-based data collection campaigns like LAPSE-RATE. The most common instruments used across all UAS platforms include barometric pressure, temperature, and relative humidity sensors. Therefore, we seek to better understand if the inter-sensor variability of commonly used sensors in atmospheric monitoring can be reduced through careful design and calibration.

The overarching objective of this work was to develop a pressure-temperature-humidity (PTH) probe designed for integration into existing UAS autopilot and data acquisition systems. Specific objectives included: (1) fabricating a set of PTH probes, (2) calibrating a set of PTH probes, and (3) validating that the calibration removes bias between individual sensors. Furthermore, we hypothesize that: (A) prior to calibration against a benchmark instrument, uncalibrated sensors will collect measurements that are significantly different for a given parameter; (B) after calibration, sensors will collect measurements that show no significant differences for a given parameter.

This manuscript provides both technical documentation of the PTH probe design in support of future use within the UAS-based precision meteorology community (Section 2.1, Section 2.2, Section 2.3, Section 2.4 and Section 3.1) and research findings on PTH probe calibration and validation (Section 2.5, Section 3.2, Section 3.3 and Section 3.4) with relevance to the broader scientific community. The novelty in our approach lies in the ability to apply zero- or first-order calibrations to the individual sensors on the PTH probe using a simple serial communication scheme. Here, we demonstrate that approach through a laboratory calibration of a set of PTH probes to remove intersensory bias and benchmark their output to a reference instrument. The process would also be useful in the field to address distributed sensing applications where sensor data from instruments currently used are calibrated after measurements have been collected [2,12,13,21]. For example, a set of PTH probes connected to a single ground station through a wireless network or via existing UAS telemetry radios could receive calibration settings to remove intersensory bias or even dynamically re-calibrate individual PTH probes when near a reference instrument such as a Mesonet station [10].

## 2. Materials and Methods

### 2.1. Sensor Selection

The primary purpose of the PTH probe was to combine pressure, temperature, and humidity measurements into a single formatted data stream to better facilitate interfacing with existing UAS control and data acquisition systems. Modern embedded sensors typically feature digital interfaces that operate using an external microcontroller or microprocessor. Several standard serial communication protocols, including Inter-Inter Circuit (I^2^C), Serial Peripheral Interface (SPI), and Universal Asynchronous Receiver-Transmitter (UART), are commonly used for embedded sensors. I^2^C and SPI are synchronous protocols that use a clock signal for timing data transfer between devices. UARTs do not include a clock signal and require communication settings between devices to match (e.g., Baud rate, data length, parity, and stop bits).

The BMP390 digital pressure sensor (Bosch Sensortec GmbH, Reutlingen, Germany), PPG101A6 thin-film platinum resistor (Littelfuse, Chicago, IL, USA) interfaced with an ADS122C04 analog-to-digital converter (Texas Instruments, Dallas, TX, USA), and an SHT40 digital humidity sensor (Sensirion, Staefa ZH, Switzerland) were selected as the environmental sensors. The BMP390 and SHT40 also featured a digital temperature sensor co-located with their barometric pressure and relative humidity sensors, respectively. All three sensors supported the I^2^C communication protocol with unique device addresses. Manufacturer specifications for all three sensors are provided in Table 1.

### 2.2. PTH Probe Design

**Figure 1:**
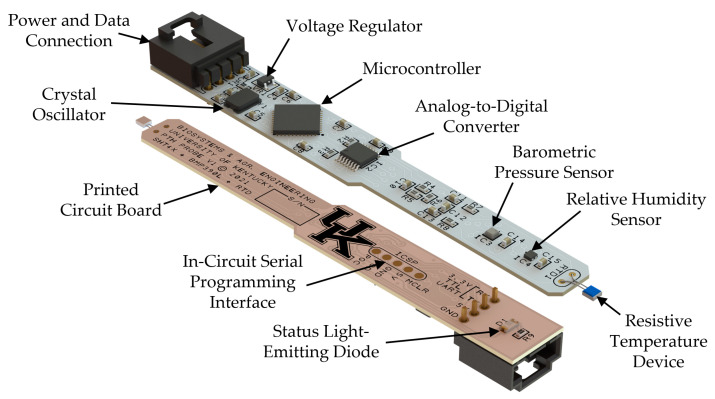
Top and bottom isometric views of the PTH probe design with main components identified.

A printed circuit board (PCB) was designed using PCB Artist (V4.0, WestDev Ltd., Aurora, CO, USA) and physically modeled in SOLIDWORKS (V2020, Dassault Systèmes, Vélizy-Villacoublay, France). Figure 1 shows a 3D rendering of the PTH probe with major components annotated. The probe was designed to operate using a 5 VDC source and communicate with a host device via a universal asynchronous receiver transmitter (UART) operated at 3.3 VDC truth table logic (TTL). Power, ground, transmit (TX), and receive (RX) entered the PCB via a 4-pin latching connector (0705530003, Molex, Lisle, IL, USA). The 5 VDC supply was regulated to 3.3 VDC using a linear voltage regulator (TPS73133DBVR, Texas Instruments, Dallas, TX, USA). The 3.3 VDC supply was used to power a microcontroller (DSPIC30F3014, Microchip, Chandler, AZ, USA) along with the pressure, temperature, and humidity sensors. The microcontroller used an external clock source provided by an 8 MHz crystal oscillator (ABM3-8.000HHZ-D2Y-T, Abracon LLC, Spicewood, TX, USA).

A two-layer design was used to route traces on the top and bottom of the PCB. The top layer was used to convey signals between components, while the bottom layer was predominately used to supply power. Roughly half of the bottom layer contained a copper pour to route ground and serve as a heatsink for the larger integrated circuits. Sensors were placed away from integrated circuits and off the ground plane to limit heat transfer from potential heat sources. Supporting components (e.g., resistors, capacitors) were located near their associated devices. All components, apart from a status light-emitting diode (LED) and its associated current-limiting resistor, were located on the top PCB layer.

The width of the PCB was constrained by the power and data connector and integrated circuits, starting at 12.7 mm and tapering to 9.5 mm roughly mid-way along the length. The length of the PCB was 95.2 mm and was constrained by the space needed to run traces between components and to facilitate rework if components needed to be removed from the PCB. The RTD sensor extended roughly 4.4 mm beyond the end of the PCB to increase exposure to ambient air and limit thermal conductivity to the PCB. The thickness of the PCB was 0.79 mm, which was selected to provide a rigid surface for mounting components while limiting mass.

### 2.3. PTH Probe Assembly

PTH probes were assembled using a two-step process. Reflow soldering was used to attach surface mount components on the top side of the PCB. The reflow soldering process involved silk-screening lead-free no-clean solder paste (GC 10, Henkel Corporation, Westlake, OH, USA) onto the top side of the PCB using a 0.1 mm thick stainless-steel stencil. Components were placed by hand before inserting the assembly in a reflow oven (MC301, Manncorp, Hatboro, PA, USA). Hand soldering was used for all through-hole components and surface mount components on the bottom side of the PCB after reflow soldering was completed. Four PTH probes were assembled per batch, and two consecutive batches were used in the subsequent calibration/validation study.

### 2.4. PTH Probe Firmware Development

**Figure 2:**
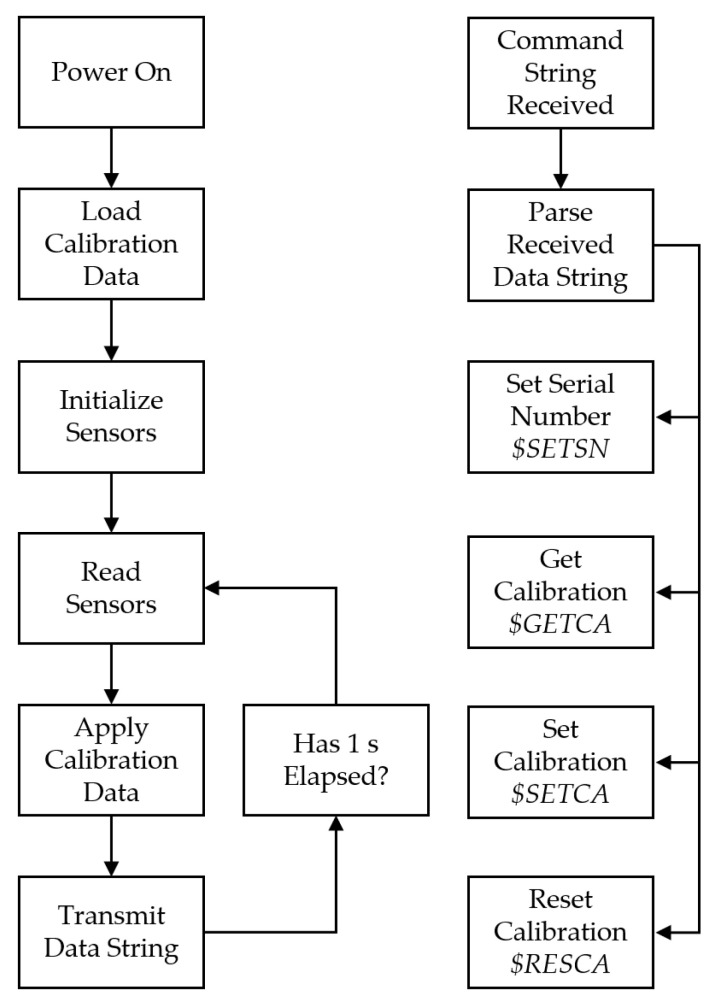
Block diagram of the PTH probe microcontroller firmware generalizing functionality. The main thread handled sensor measurements, data processing, and data transmission. An interrupt service routine handled serial data input from a host device for setting the serial number, retrieving existing calibration data, setting the calibration data to specific values, and resetting the calibration data to factory defaults.

The microcontroller served as the interface between individual sensors and a host device (i.e., UAS flight controller or data acquisition system). An integrated development environment (MPLAB IDE V8.70, Microchip) and C-language compiler (MPLAB C30, Microchip) were used to write the firmware on the microcontroller. Figure 2 shows a block diagram of the PTH probe microcontroller firmware.

The PTH probe began operating when a nominal 5 VDC was supplied to the power and data connector. The microcontroller’s internal hardware peripherals were configured in preparation for serial communication during the power on phase. Next, linear calibration data for each sensor were loaded from non-volatile EEPROM. By default, calibration settings for all sensors were assigned a slope of one and offset of zero when initially programmed. These calibration parameters were subsequently updated after sensor calibration was completed. Next, the BMP390 digital pressure sensor and ADS122C04 analog-to-digital converter used in the RTD circuit were initialized. The BMP390 was configured to operate in “ultra-high resolution” mode, and the individual calibration data needed to convert raw measurements from integer parameters to floating-point pressure in units of Pascals were read. The ADS122C04 was configured to operate as a 2-wire RTD by supplying a 500 µA current through the PPG101A6 platinum resistor and measure the differential voltage across the resistor. The SHT40 did not require any initialization. Data from the pressure, temperature, and humidity sensors were successively read through the I^2^C bus. Calibration data were applied to individual sensor measurements, and the resulting values were packaged into a formatted string before being transmitted out the UART. The measurement calibration-transmission process was repeated at 1 Hz through an internal hardware timer.

**Figure 3:**
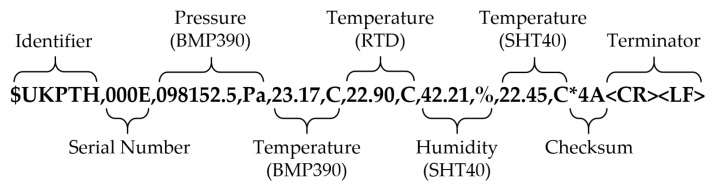
Example formatted string transmitted from the PTH probe to the host at 1 Hz over 3.3 VDC TTL UART. The checksum was the bit-wise exclusive OR of all 8-bit ASCII characters between the ‘$’ and ‘*’ characters and displayed as a 2-digit hexadecimal number. The terminator comprised the non-printable carriage return <CR> and line feed <LF> characters.

The serial data transmitted from the PTH probe’s UART followed a fixed-format comma-delimited scheme and included an identifier, serial number, environmental measurements with associated engineering units, checksum, and string terminator (Figure 3). The identifier was used to distinguish which type of string was being transmitted. The serial number was a 4-digit hexadecimal value used to distinguish individual PTH probes and their associated calibration data. Each subsequent environmental measurement was accompanied by its associated engineering units to mitigate ambiguity (e.g., °C vs. °F). An asterisk character was used to separate the checksum from the rest of the serial data. The checksum was calculated as the bit-wise exclusive OR of all 8-bit ASCII characters between, but not including, ‘$’ and ‘*’ and displayed as a 2-digit hexadecimal number. The final two characters in each string were always ASCII 0x0D and 0x0A, representing non-printable carriage return <CR> and line feed <LF>. These terminating characters facilitated data-logging in the PTH probe’s native serial data output format. Incoming data written directly to a text (*.txt) or comma-separated-values (*.csv) file were automatically formatted with each PTH probe measurement on a separate line using the data output scheme.

**Figure 4:**
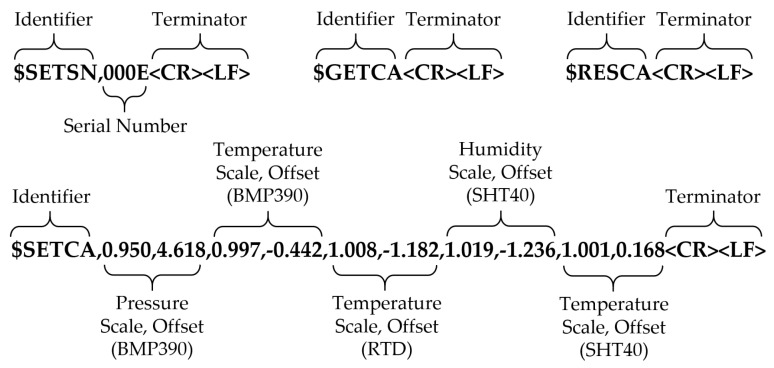
Example formatted strings received by the PTH probe from the host over 3.3 VDC TTL UART. The PTH probe responded by echoing the string with relevant data and checksum inserted before the terminator.

Serial data received by the PTH probe UART followed a similar scheme with some minor differences depending on the data being received. The microcontroller buffered all incoming UART characters and searched the buffer for ‘$’ and <LF> characters. If found in order, all characters between and including were moved from the buffer and flagged as a new message. This process was accomplished in hardware using the UART RX interrupt service routine to prevent received data from being lost. Four message types were supported to handle tasks after the microcontroller firmware had been loaded. Serial numbers were assigned using a Set Serial Number ($SETSN) message. The current calibration data was retrieved using a Get Calibration ($GETCA) message. New calibration data were applied using the Set Calibration ($SETCA) message. Finally, existing calibration data were reset to an offset of zero and a slope of one using a Reset Calibration ($RESCA) message. The exact data format with example parameters are shown in Figure 4.

### 2.5. Calibration System Development

**Figure 5:**
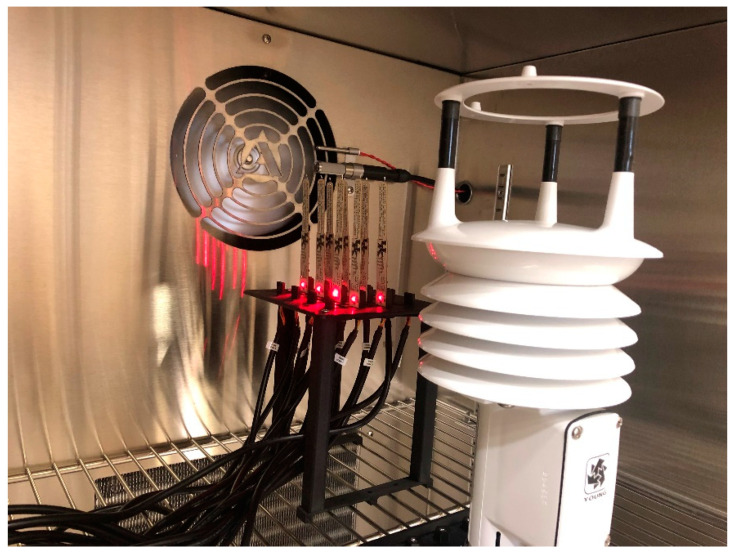
Eight PTH Probes in an environmental chamber. The environmental chamber’s temperature and relative humidity sensors and the weather station’s barometric pressure sensor were used as reference instruments for calibration and validation. The weather station’s ultrasonic anemometer also confirmed that airflow exceeded 1.0 m/s to achieve manufacturer-specified sensor response times.

An environmental chamber (LHE-6, Associated Environmental Systems, Acton, MA, USA) with panel mount controllers (EZ-ZONE PM 1/16 DIN, Watlow, St. Louis, MO, USA) for temperature and humidity control was connected via USB-to-RS-485 adapter (ULINXTM 485USBTB-2W, Advantech B+B SmartWorx, Ottawa, IL, USA) to a computer. The PTH probes were connected to the computer with UART-to-USB cables (TTL-232R-3V3, FTDI, Glasgow, UK) through a USB hub (ID-US0611-S1, SIIG, Hayward, CA, USA) and mounted inside the environmental chamber in an upright orientation, positioned at a similar height to a weather station (92,000, R.M. Young, Traverse City, MI, USA) which contained the barometric pressure sensor used as a reference instrument for calibration and an ultrasonic anemometer to verify minimum airflow requirements to meet manufacturer specified sensor response times. Figure 5 shows the PTH probes and weather station installed in the environmental chamber.

**Figure 6:**
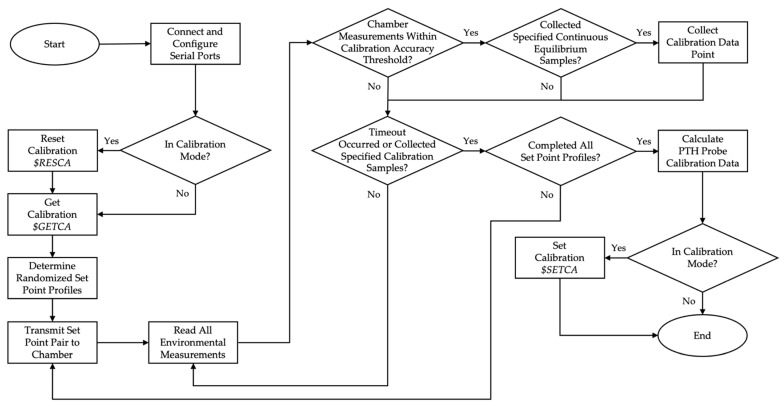
Block diagram of the automated calibration system generalizing functionality.

The App Designer within MATLAB (R2021b, MathWorks, Natick, MA, USA) was used to write the program that runs the automated calibration process for the PTH probes. Figure 6 shows a block diagram of the automated calibration program.

At the start, the user specified the serial ports of both the environmental chamber and weather station, as well as any serial ports that were to be excluded from the calibration process. If the specified serial ports for either the environmental chamber or weather station were not found, a warning was raised, and the user was requested to reinput the information. Otherwise, the serial ports were connected to and configured. RS-485 settings for the environmental chamber were 38,400 Baud, 8 data bits, no parity, and 1 stop bit. The terminator was comprised of the non-printable carriage return <CR>. RS-232 settings for the weather station were 9600 Baud, 8 data bits, no parity, and 1 stop bit. The terminator was comprised of the non-printable carriage return <CR>.

The temperature and relative humidity calibration setpoint thresholds were also defined by the user, along with the required number of continuous samples within the threshold to assume equilibrium and the number of samples to collect for calculating the calibration data. Additionally, the user specified whether the data collection was for calibration or validation purposes. If calibration mode was selected, the existing calibration data were reset to factory defaults using a Reset Calibration ($RESCA) message. Otherwise, the calibration data for the PTH probes remained as it was. Regardless of data collection mode, the current calibration data was then retrieved using a Get Calibration ($GETCA) message.

The desired temperature and relative humidity set point profiles were retrieved from a user-defined Excel file. A list of all possible combinations of temperature and relative humidity pairs was determined, randomized, and filtered to exclude set point combinations outside the standard humidity range or below the dew point inside the environmental chamber. A set point pair was communicated to the environmental chamber panel mount controllers via Modbus Remote Terminal Unit (RTU) protocol. After which, the ASCII serial data transmitted from the weather station and each PTH probe UART was sequentially read and parsed. The actual temperature and relative humidity values of the environmental chamber were read via the Modbus RTU protocol.

The measurement process was repeated without further action until both the environmental chamber temperature and relative humidity measurements fell within the defined calibration setpoint thresholds. At which point, if the specified number of continuous samples within the threshold was met, the environmental measurements were recorded as data points to use for calculating the calibration data. There was a timeout period implemented to prevent an indefinite loop in the case of the environmental chamber measurements not reaching equilibrium within the specified calibration setpoint threshold, where that setpoint pair was then omitted from calibration calculations.

Once the required number of samples was recorded, the next setpoint pair was sent to the panel mount controllers, and the measurement process was repeated until enough samples within the calibration accuracy threshold were detected and all setpoint pairs had been communicated to the environmental chamber.

The calibration data for each PTH probe were then calculated using a first-order polynomial regression employing the least-squares method. If calibration mode had been selected, the new calibration data were packaged and applied to each PTH probe using the Set Calibration ($SETCA) message.

### 2.6. Calibration

Eight PTH probes assembled in two batches of four probes were calibrated concurrently. Table 2 specifies the temperature and relative humidity setpoints input into the automated calibration program during calibration. A total of 100 setpoint combinations were determined, with 26 pairs excluded due to falling outside the limits of the standard humidity range for the environmental chamber.

Table 3 provides the calibration setpoint thresholds and sample size requirements used when collecting the calibration dataset. The setpoint thresholds and time-out period were determined through previous calibration runs during the calibration routine development to allow for as many of the setpoint pairs to be reached within a reasonable amount of time.

After applying the least-squares regression to the collected calibration dataset, five pairs of calibration coefficients, a slope and an offset corresponding to each sensor, were generated for each PTH probe. These calibration data were packaged and sent to the corresponding PTH probe.

### 2.7. Validation

With the calibration data programmed to each PTH probe, the validation process used a different set of temperature and relative humidity setpoints within the same range as the calibration set. Table 4 specifies the temperature and relative humidity setpoint profiles that were input into the automated calibration program during validation. In this case, 91 setpoint combinates were determined, with 25 pairs excluded from the onset due to not being feasible given the environmental chamber specifications. The same setpoint thresholds and sample sizes shown in Table 3 for the calibration process were used during the validation process.

The outcomes of the validation process again yielded five pairs of regression coefficients, a slope and an offset corresponding to each sensor, for all PTH probes. However, the slopes were now expected to be 1, and the offsets 0, which would demonstrate that the previously applied calibration removed bias in the PTH probe sensors.

### 2.8. Statistical Analysis

Statistical analysis was performed with MATLAB and SAS (Version 9.4, SAS Institute, Cary, NC, USA). Calibration and validation data were grouped by PTH probe serial number, sensor, and set point. The setpoint was defined as a random effect to account for the variability at different set points not associated with sensors, considered a fixed effect. The assumptions of normality and homogeneous variances were checked prior to analysis. The SHT40 relative humidity data after calibration was logarithmically transformed, and all other data met the assumptions. Statistical significance was assumed at *p*-value < 0.05. When significant differences were detected, Tukey-Kramer post-hoc multiple comparisons analysis was performed to determine which sensors differed.

## 3. Results

### 3.1. PTH Probe Specifications

**Figure 7:**
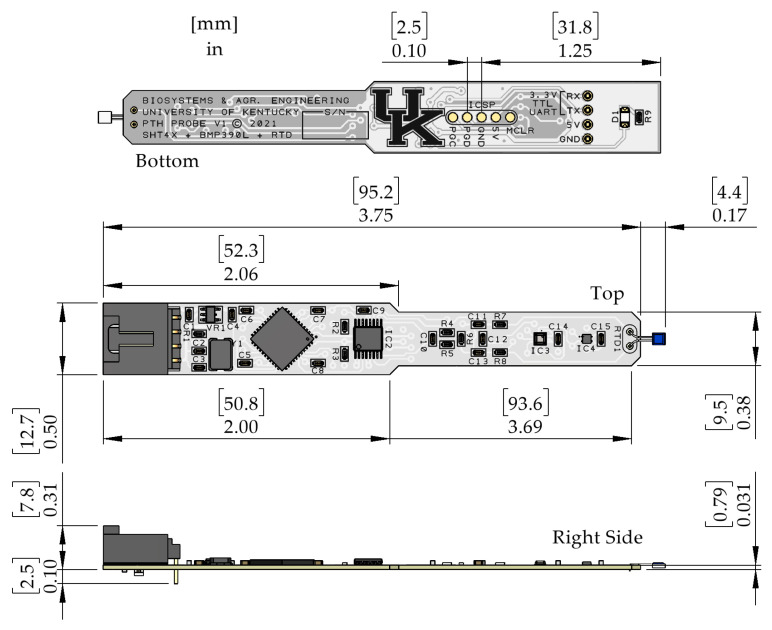
Dimensioned drawing of the PTH probe. Units are shown in [millimeters] and inches.

Figure 7 provides the mechanical dimensions of the PTH probe view from the bottom, top, and right sides. Dimensions on the bottom view show the distance from the edge of the PCB to the center pin on the in-circuit serial programmer (ICSP) interface and the spacing between pads. The top view shows the dimensions of the PCB and the typical distance that the RTD platinum resistor extends beyond the PCB. The side view shows the PCB thickness and the distance that the power and data connector extend above and below the bottom of the PCB.

Table 5 summarizes the PTH probe operating characteristics. While the PTH probe was designed to operate from a nominal 5 VDC source (V_s_), the linear voltage regulator used to supply 3.3 VDC (V_r_) to all downstream components allowed for a range of input voltages with negligible change in PTH probe performance.

The RTD circuit can accommodate any two-wire 100 Ω through-hole platinum resistor with leads of 0.5 mm (0.02 in) in diameter or smaller. The RTD circuit was driven using a constant 0.5 mA current source (I_RTD_), which consumed roughly half of the total supply current (I_s_). The PPG101A6 platinum resistor used in the PTH probe had a nominal dissipation constant of P_D_ = 1.8 mW/°C, which defines the amount of power required to self-heat the resistor by one degree Celsius above ambient temperature. The nominal dissipation constant assumes standard temperature and 1 m/s airflow. The rise in temperature above ambient (ΔT) due to the power dissipated in the PPG101A6 was estimated using the following equation:

(1) $$\Delta T=\frac{I_{\mathrm{RTD}}{}^{2}R_{\mathrm{RTD}}}{P_{D}}=\frac{0.025\;\mathrm{mW}\;}{1.8\;\mathrm{mW}/{}^{\circ}C}=0.014\;{}^{\circ}C$$

and was negligible when compared to the expected accuracy of the RTD circuit.

The data output rate (F_s_) was set to 1 Hz using a hardware timer interrupt service routine in the PTH probe firmware. While it was possible to sample individual sensors at a rate of 10 Hz or faster, processing raw measurements limited the output rate. Sampling the sensors, applying floating-point calibration data, and transmitting the serial data string using the formatted print function took slightly longer than half a second when operating the DSPIC30F3014 with a 2 MHz instruction clock.

The operating pressure (P), temperature (T), and relative humidity (H) were defined by the range of environmental conditions allowable for all components on the PTH probe. This resulted in the temperature range for the RTD sensor being substantially reduced and the relative humidity being limited to non-condensing environments. Condensation within the SHT40 or BMP390 would produce an erroneous output. However, these specifications encompass a reasonable range of environmental conditions expected when collecting atmospheric measurements using a UAS.

### 3.2. Calibration/Validation System

**Figure 8:**
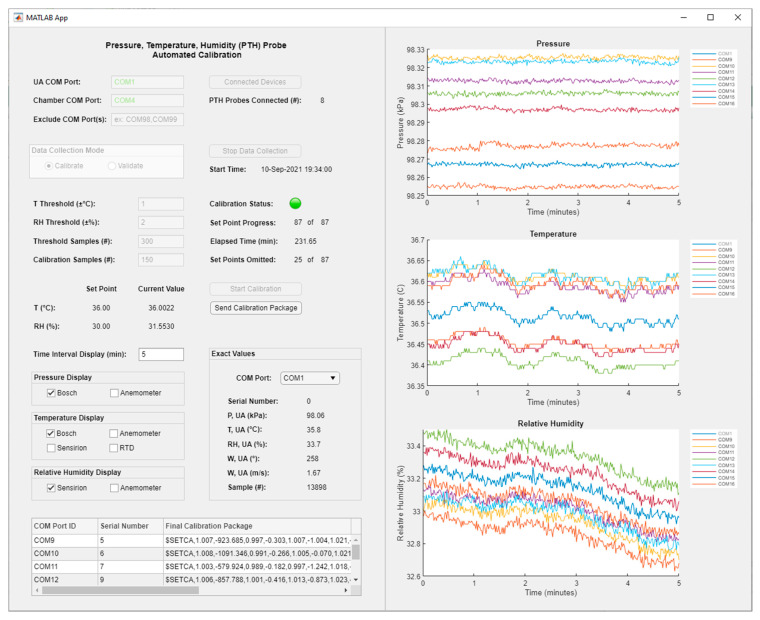
Automated calibration program graphical user interface developed in MATLAB App Designer environment.

Figure 8 shows the graphical user interface (GUI) of the automated calibration program. The graphical displays included three separate plots for pressure, temperature, and relative humidity with adjustable visibility options for measurements from the different sensors on the PTH probes and the weather station. While the time interval for the plots defaulted to displaying the most recent five minutes of data, which was approximately equivalent to 300 data samples, it was able to be adjusted by the user throughout the calibration/validation run. Included in the display was also a drop-down menu comprised of a list of the weather station and PTH probe serial ports. When a serial port was selected, the exact measurement values for the corresponding component were updated on the interface.

### 3.3. Calibration

**Figure 9:**
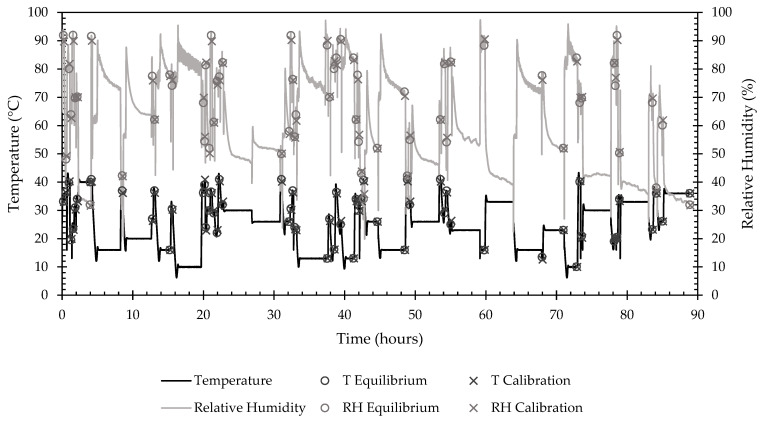
Time response of environmental chamber settings for temperature and relative humidity during a calibration run, with additional points of interest for the start of equilibrium threshold and calibration data collection periods. Lines show the temperature and relative humidity profiles over the duration of the calibration. O’s show when the chamber reached equilibrium and calibration data collection began. X’s show when calibration data collected ended.

The calibration run took 88.99 h to complete a temperature and relative humidity set point profile with 74 randomized set point combinations that fell within the environmental chamber’s capability. Figure 9 depicts the time response of the environmental chamber throughout the calibration process. Of the 74 pairs, 12 did not reach equilibrium within the specified timeout period of 4 h and were therefore excluded from the calibration calculations.

**Figure 10:**
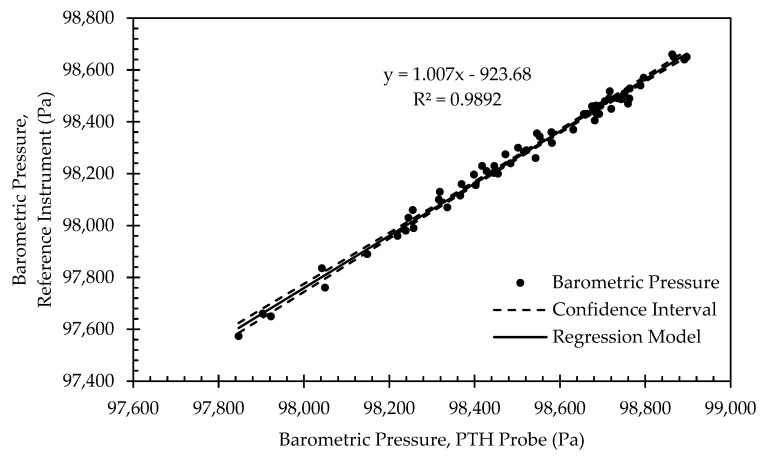
Regression model and corresponding confidence interval for barometric pressure calibration data from representative PTH probe (SN: 0x0005).

**Figure 11:**
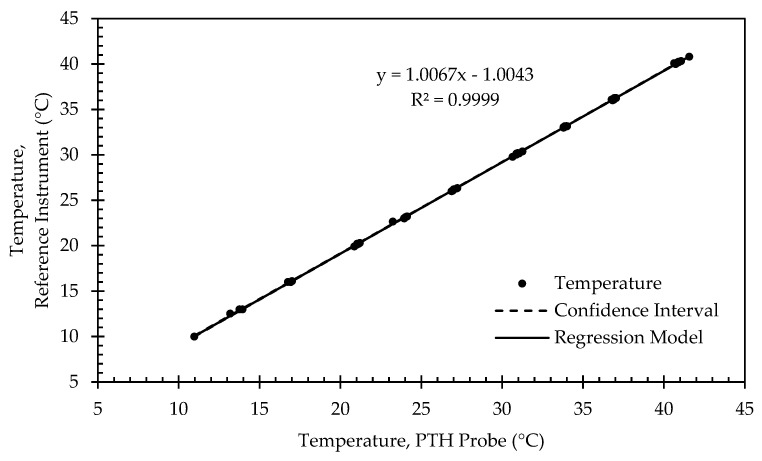
Regression model and corresponding confidence interval for temperature calibration data from representative PTH probe (SN: 0x0005).

**Figure 12:**
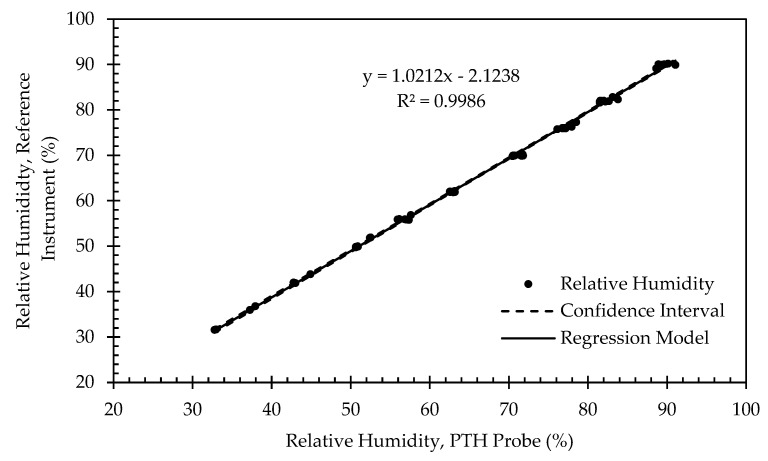
Regression model and corresponding confidence interval for relative humidity calibration data from representative PTH probe (SN: 0x0005).

Example calibration data for a single PTH probe are shown in Figure 10, Figure 11 and Figure 12. Figure 10 presents example initial calibration results for the barometric pressure sensor. While the linear regression model proved to be a good fit, offset from the linear regression exceeds the expected absolute accuracy of the BMP390. This was likely due to the limited range of samples, roughly 1000 Pa, which only represented 1% of the full-scale output range of the sensor. Limited range combined with the distance to the intercept meant that small changes in the slope of the linear regression resulted in a large offset. All eight PTH probes exhibited a similarly large offset. Thus, the linear regression was deemed unsuitable for the barometric pressure calibration data, and a 1-point calibration was applied using the average offset between the PTH probe and the reference instrument. The offset varied in magnitude between 236.9 and 307.6 Pa for all PTH probes (Table 6).

Figure 11 presents example calibration results for the RTD sensor. Calibration points were clustered near the target setpoints with some deviation due to the 1 °C tolerance threshold allowed for determining when to begin collecting calibration data. As expected, the relationship between the RTD temperature output and the reference instrument was highly linear (R^2^ > 0.99). The slope was nearly 1, and there was a 1 °C bias in temperature for this particular PTH probe. The large bias was not surprising, given the uncalibrated temperature output did not consider actual component values used in the RTD amplifier circuit. The offset varied in magnitude between 0.065 °C and 1.242 °C for all PTH probes (Table 6).

Figure 12 presents example calibration results for the relative humidity sensor. Similar to the RTD results, relative humidity calibration points were clustered near the target setpoints but with additional scattering due to the larger 2% RH tolerance threshold. The relationship between the relative humidity sensor and the reference instrument was highly linear (R^2^ > 0.99). The slope was nearly 1, and the offset fell within the typical accuracies of the SHT40 and the reference instrument. The offset varied in magnitude between 1.540% and 2.655% for all PTH probes (Table 6).

The regression coefficients for all eight uncalibrated PTH probes are shown in Table 6. Slopes of the linear regressions were close to 1 across all sensors. Offsets varied but were still within the maximum accuracy specifications of each sensor, with the RTD exhibiting the largest change in offset across all three sensors’ temperature outputs. Offsets for the BMP390 barometric pressure sensor were consistent across all sensors, whereas the other sensors exhibited higher variance. This can be explained in part by BMP390, including unique factory parameters used when generating the output for each sensor rather than a fixed set of parameters for all sensors.

The lower and upper bounds of the 95% confidence intervals for the linear regression coefficients are shown in Table 7. These intervals indicate the uncertainty in slope and offset of the linear regression resulting from a limited set of calibration points. A tight confidence interval indicates the sample size adequately represented the population. The only sensor to produce a large confidence interval during calibration was the BMP390 barometric pressure sensor, and that linear regression was discarded in favor of a 1-point calibration.

The performance of the regression models used in calibration is shown in Table 8. The coefficient of determination (R^2^) and root mean square error (RMSE) are shown for all sensors except the BMP390 barometric pressure sensor. R^2^ indicates how well the PTH probe measurements are replicated by the model. In all instances, the model was a good fit and produced R^2^ values near 1. RMSE represents the average deviation of the residuals between the PTH probe measurements and the regression model. RMSE is represented in the same units as its associated parameter. RMSE for the SHT40 relative humidity sensor varied between 0.622% and 0.787%. RMSE for the RTD and the temperature sensors internal to the BMP390 and SHT40 for each probe were similar, and varied between 0.054 °C and 0.143 °C. RMSE for the BMP390 barometric pressure sensor, calculated directly from the PTH probe and reference instrument, varied from 238.350 Pa to 308.586 Pa. One noteworthy trend emerged in RMSE for all sensors. The PTH probes were mounted in two rows in front of the circulation fan inside the environmental chamber. PTH probes closest to the fan, where the environmental chamber’s reference temperature and humidity sensors were located, exhibited the highest RMSE. RMSE decreased as the distance from the fan increased. This was an unexpected result as we assumed homogeneity inside the chamber. Air exchange between the chamber and the outside environment that cabling passed through was likely the cause of this temperature and humidity gradient.

Analyses for intersensory bias in uncalibrated PTH probes are shown in Table 9. From the Tukey-Kramer multiple comparisons procedure, all PTH probes demonstrated significant differences between the uncalibrated sensors. The BMP390 barometric pressure and RTD temperature sensors were both shown to have seven statistically different groups. The SHT40 relative humidity sensor had six distinct groups, the BMP390 temperature sensor had five, and the SHT40 temperature sensor had four. However, the resulting least-squares mean estimates, useful for testing linear contrasts among predictions, indicate relatively low variability in the collected data.

### 3.4. Validation

**Figure 13:**
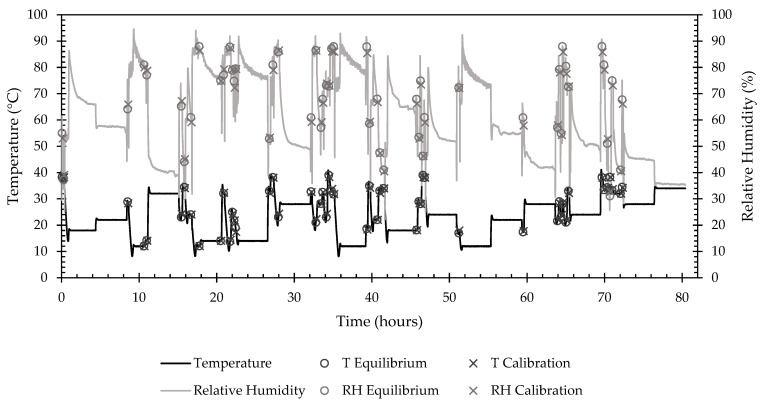
Time response of environmental chamber settings for temperature and relative humidity during validation run, with additional points of interest for the start of equilibrium threshold and calibration data collection periods. Lines show the temperature and relative humidity profiles over the duration of the validation. O’s show that when the chamber reached equilibrium and validation, data collection began. X’s show when validation data collected ended.

The validation run took 80.38 h to complete a temperature and relative humidity setpoint profile with 66 setpoint combinations (Figure 13). Of the 66 pairs, 15 did not reach equilibrium within the specified timeout period and were excluded from the calibration calculations.

**Figure 14:**
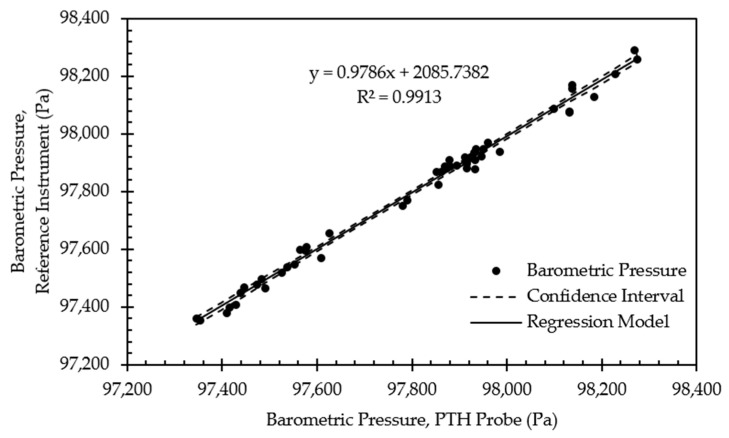
Regression model and corresponding confidence interval for barometric pressure validation data from representative PTH probe (SN: 0x0005).

**Figure 15:**
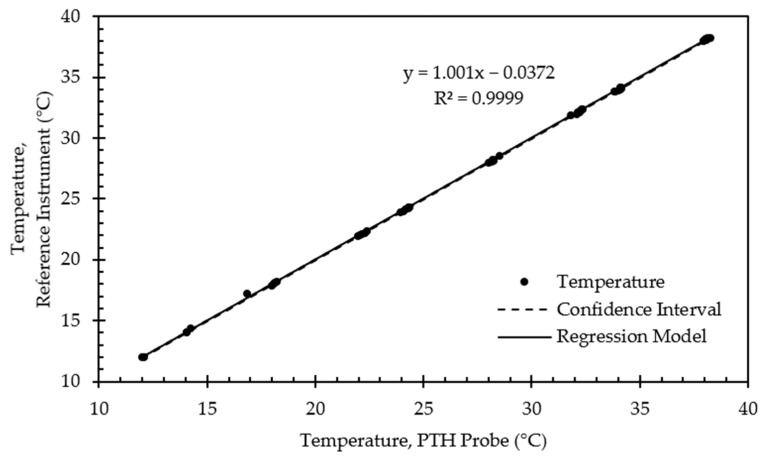
Regression model and corresponding confidence interval for temperature validation data from representative PTH probe (SN: 0x0005).

**Figure 16:**
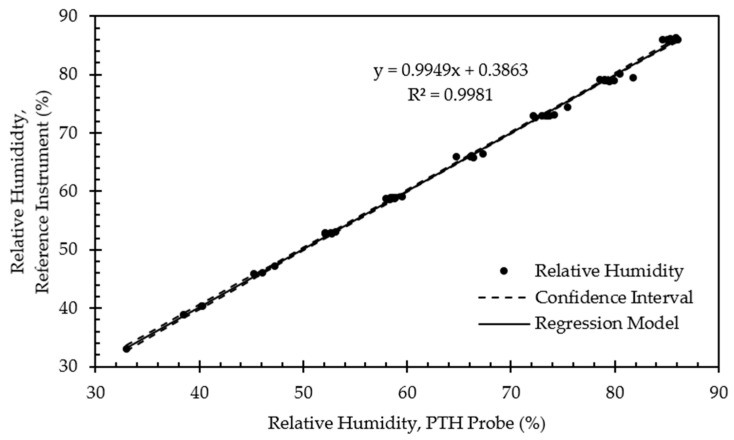
Regression model and corresponding confidence interval for relative humidity validation data from representative PTH probe (SN: 0x0005).

Example validation data for a single PTH probe are shown in Figure 14, Figure 15 and Figure 16. Figure 14 presents example initial validation results for the barometric pressure sensor. The linear regression was again deemed unsuitable for the barometric pressure calibration data due to the limited range of samples which only represented 1% of the full-scale output range of the sensor. As such, the average offset between the PTH probe and the reference instrument was used to assess performance. The offset varied in magnitude between 1.7 Pa and 4.5 Pa for all PTH probes, two orders of magnitude less than the offsets from the calibration run (Table 10).

Figure 15 presents example validation results for the RTD sensor. Validation points were similarly clustered near the target setpoints with some deviation due to the 1 °C tolerance threshold allowed for calibration data collection. The relationship between the RTD temperature output and the reference instrument remained highly linear (R^2^ > 0.99). The slope was nearly 1, and there was a 0.04 °C bias in temperature for this particular PTH probe, which was reduced from its uncalibrated bias of 1 °C. The offset varied in magnitude between 0.004 °C and 0.138 °C for all PTH probes (Table 10). 

Figure 16 presents example validation results for the relative humidity sensor. Relative humidity calibration points were again clustered near the target setpoints with additional scattering due to the 2% RH tolerance threshold allowed for validation data collection. The relationship between the relative humidity sensor and the reference instrument remained highly linear (R^2^ > 0.99). The slope was nearly 1, and the offset was further reduced and maintained within the typical accuracies of the SHT40 and the reference instrument. The offset varied in magnitude between 0.306% and 0.468% for all PTH probes (Table 10).

The regression coefficients for all eight calibrated PTH probes are shown in Table 10. Similar to the calibration run results, slopes of the linear regressions were close to 1 across all sensors, and offsets were still within maximum accuracy specifications.

Table 11 displays the lower and upper bounds of the 95% confidence intervals for the linear regression coefficients from the validation run. These intervals indicated low variability and a small margin of error across all PTH probes. The confidence intervals for the BMP390 barometric pressure sensor were discarded due to using a 1-point calibration in place of linear regression.

Performance metrics, such as coefficient of determination (R^2^) and root mean square error (RMSE), of the regression models used in the validation run are shown in Table 12. In all instances, the regression model was a good fit and produced R^2^ values close to 1. RMSE for the SHT40 relative humidity sensor varied between 0.638% and 0.779%. RMSE for the RTD and the temperature sensors internal to the BMP390 and SHT40 for each probe were similar and varied between 0.061 °C and 0.126 °C. RMSE for the BMP390 barometric pressure sensor varied from 22.536 Pa to 30.141 Pa and demonstrated the largest decrease compared to the calibration run. A similar trend was observed in which RMSE decreased as the distance from the circulation fan within the environmental chamber increased.

Analyses for intersensory bias in calibrated PTH probes are shown in Table 13. The Tukey-Kramer multiple comparisons procedure demonstrated a reduction in the significant differences between sensors. The BMP390 barometric pressure sensor went from seven statistically different groups to two, while the SHT40 relative humidity sensor went from six to three. All temperature sensors, which previously contained four to seven distinct groups, were now found to have no statistical differences between each other. In most cases, the difference between the least-squares means used in the analysis was now detected in the third or fourth significant figure of the estimate, indicating reduced variability within the data collected after calibration was applied. The least-squares means also showed decreased variability in estimates between sensors taking redundant measurements across the same probe.

## 4. Discussion

A set of eight PTH probes were fabricated and calibrated for pressure, temperature, and relative humidity using an environmental chamber and weather station as reference instruments. A validation procedure was then used to evaluate if the applied calibration successfully reduced intersensory bias. The relative humidity range of the setpoint profiles used for this experiment was a good representation of the values encountered in field work. However, the temperature range was limited by the specifications of the environmental chamber and therefore did not include temperatures below freezing, which would be a reasonable condition to encounter seasonally or during field work at high altitudes.

Based on the results of the Tukey-Kramer procedures, fewer calibrated sensors were determined to be statistically different from each other compared to the uncalibrated sensors. Small variability within the calibrated results was still statistically significant; however, this was primarily due to the large sample size and low variance of the collected data. As such, it was determined that despite some sensors continuing to register as statistically different, those significant differences are not important in a practical sense. However, if an application required strict uniformity between sensors with no statistical difference, then the calibrated probes could be sub-selected based on the Tukey-Kramer results to ensure those requirements are met.

The results justify the need for calibrating the PTH probes rather than relying on nominal outputs. The linear regression offsets are particularly useful for quantifying the difference in pre- and post-calibrated PTH probe outputs. The offsets produced in the linear regression models demonstrate the bias between identical sensors before calibration (Table 6) and the extent to which that bias was minimized after calibration (Table 10). For example, the range in barometric pressure offset was reduced from 70.7 Pa to 2.8 Pa. In the absence of a benchmark, the sensor-to-sensor variability exceeded the manufacturer-specified maximum accuracy of ±50 Pa before calibration, and fell within the manufacturer-specified typical accuracy of ±3 Pa after calibration. Thus, calibration was needed to meet the desired accuracy specification.

The LSMs presented before calibration (Table 9) and after calibration (Table 13), along with the Tukey-Kramer groupings, tell a similar story. LSM is the average of the means for all calibration data. The range in LSMs across all temperature sensors was reduced from 1.658 °C to 0.013 °C. Some discrepancy between different sensor models should be reasonably expected given different manufacturers, but the substantial reduction in variability demonstrates the value of calibrating different sensor models to a common reference.

Future work should expand the calibration procedure to include a separate process for barometric pressure calibration. In this work, the temperature and relative humidity calibrations were obtained through a linear regression model, while barometric pressure required an adjusted 1-point calibration. A distinct barometric pressure calibration procedure would provide a greater calibration range and allow for an improved linear regression model to be used.

Additionally, there is further work to be done with the calibration/validation system developed for automated calibration data collection. For this experiment, the MATLAB-based system was structured to collect data sequentially. As such, there were limitations on scaling up the number of PTH probes that could be calibrated simultaneously. Re-structuring to a parallel environment could help address this scaling issue for future calibration procedures, as well as provide better intersensory comparisons between probes.

## 5. Conclusions

The overarching objective of this work to develop a PTH probe was accomplished through achieving the specific objectives of (1) fabricating, (2) calibrating, and (3) validating a set of PTH probes. Our working hypothesis that (A) uncalibrated sensors would exhibit statistically significant differences while (B) calibrated sensors would not exhibit statistically significant differences was mostly supported by the results. Specifically, all temperature sensors were shown to not be significantly different after calibration. The differences in barometric pressure and humidity sensors after calibration were negligible for the designed purposes of the PTH probe. Therefore, we conclude that the objectives of this work were successfully met.

## 6. Patents

A provisional patent titled “Systems and Methods for UAS-Based Distributed Atmospheric Monitoring” based upon this work was submitted to the US Patent Office (Application #63290918).

## Figures and Tables

**Table 1:** Nominal specifications for pressure, temperature, and relative humidity sensors provided by sensor manufacturers. Maximum accuracy encompasses the entire operating range, while typical accuracy is either a limited range or at standard ambient pressure/temperature/humidity.

Sensor	Parameter	Units	Operating Range	Maximum Accuracy	Typical Accuracy	Resolution	Response Time (s)
BMP390	Barometric Pressure	Pa	30,000 to 125,000	±50	±3	0.17	0.005 ^3^
	Temperature	°C	−40 to 85	±1.5	-	0.0003	-
RTD ^1^	Temperature	°C	−200 to 600 ^2^	-	±0.15	0.001	1.2 ^4^
SHT40	Relative Humidity	%	0 to 100	±5.0	±1.8	1	6 ^4^
	Temperature	°C	−40 to 125	±1.0	±0.2	0.01	2 ^4^

^1^ RTD comprises a PPG101A6 platinum resistor and an ADS122C04 analog-to-digital converter; ^2^ Operating range is for the PPG101A6 sensor only; ^3^ Derived from maximum sampling rate; ^4^ 63% of step-change in 1.0 m/s moving air.

**Table 2:** Temperature and relative humidity setpoints used to create the calibration dataset. Ten temperature setpoints and ten relative humidity setpoints resulted in 100 setpoint pairs.

Temperature (°C)	Relative Humidity (%)
10	30
13	36
16	42
20	50
23	56
26	62
30	70
33	76
36	82
40	90

**Table 3:** Input parameters for the setpoint threshold and sample size.

Setpoint Threshold		Sample Size	
Temperature (°C)	Relative Humidity (%)	Equilibrium	Calibration
±1	±2	300	150

**Table 4:** Temperature and relative humidity setpoints used to create the validation dataset. Nine temperature setpoints and nine relative humidity setpoints resulted in 81 setpoint pairs.

Temperature (°C)	Relative Humidity (%)
12	33
14	39
18	46
22	53
24	59
28	66
32	73
34	79
38	86

**Table 5:** PTH probe operating characteristics.

Parameter		Test Condition	Min	Typ	Max	Units
V_s_	Supply Voltage	-	3.6	5.0	5.5	V
I_s_	Supply Current	V_s_ = 5.0 V	-	-	1	mA
V_r_	Regulated Voltage	3.6 < V_s_ < 5.5	3.28	3.3	3.32	V
R_RTD_	RTD Nominal Resistance	-	-	100	-	Ω
I_RTD_	RTD Drive Current	-		0.500	-	mA
F_s_	Data Output Rate	-	-	1.000	-	Hz
P	Operating Barometric Pressure	-	30	-	125	kPa
T	Operating Temperature	-	−40	-	125	°C
H	Operating Relative Humidity ^1^	-	0	-	100	%
M	PTH probe Mass	-	-	3.41	-	g

^1^ Non-condensing.

**Table 6:** Regression model coefficients for uncalibrated PTH probes.

Serial Number	BMP390				RTD		SHT40			
	Barometric Pressure (Pa)		Temperature (°C)		Temperature (°C)		Relative Humidity (%)		Temperature (°C)	
	Slope ^1^	Offset	Slope	Offset	Slope	Offset	Slope	Offset	Slope	Offset
0x0005	1.000	−236.9	0.997	−0.303	1.007	−1.004	1.021	−2.124	1.003	0.215
0x0006	1.000	−303.2	0.991	−0.266	1.005	−0.070	1.021	−1.965	0.998	0.351
0x0007	1.000	−302.2	0.989	−0.182	0.998	−1.242	1.018	−1.861	0.993	0.420
0x0008	1.000	−307.6	0.988	−0.192	0.995	−0.774	1.016	−1.691	0.989	0.505
0x0009	1.000	−291.9	1.001	−0.417	1.013	−0.873	1.023	−2.655	1.007	0.139
0x000A	1.000	−278.8	1.001	−0.459	1.013	−0.344	1.022	−2.462	1.003	0.239
0x000B	1.000	−258.4	0.996	−0.340	1.009	0.065	1.020	−2.178	0.999	0.305
0x000C	1.000	−263.4	0.991	−0.263	1.002	−0.963	1.015	−1.540	0.994	0.384

^1^ Manually set to 1.000 for 1-point calibration.

**Table 7:** Lower and upper bounds of the 95% confidence interval for calibration coefficients.

Serial Number	BMP390				RTD		SHT40			
	Barometric Pressure (Pa)		Temperature (°C)		Temperature (°C)		Relative Humidity (%)		Temperature (°C)	
	Slope ^1^	Offset ^1^	Slope	Offset	Slope	Offset	Slope	Offset	Slope	Offset
0x0005	-	-	0.994	−0.371	1.004	−1.080	1.011	−2.822	1.000	0.146
	-	-	0.999	−0.236	1.009	−0.929	1.031	−1.426	1.005	0.283
0x0006	-	-	0.989	−0.343	1.002	−0.150	1.010	−2.696	0.996	0.273
	-	-	0.994	−0.188	1.008	0.010	1.031	−1.235	1.001	0.428
0x0007	-	-	0.986	−0.280	0.994	−1.351	1.006	−2.691	0.990	0.321
	-	-	0.992	−0.084	1.001	−1.134	1.030	−1.031	0.997	0.520
0x0008	-	-	0.985	−0.301	0.991	−0.907	1.003	−2.569	0.985	0.390
	-	-	0.992	−0.083	0.999	−0.642	1.028	−0.813	0.993	0.620
0x0009	-	-	0.999	−0.466	1.011	−0.933	1.013	−3.360	1.005	0.087
	-	-	1.003	−0.367	1.015	−0.812	1.033	−1.950	1.009	0.191
0x000A	-	-	0.999	−0.514	1.010	−0.408	1.012	−3.167	1.001	0.182
	-	-	1.003	−0.405	1.015	−0.281	1.032	−1.757	1.005	0.297
0x000B	-	-	0.993	−0.410	1.006	−0.013	1.009	−2.935	0.996	0.230
	-	-	0.998	−0.269	1.011	0.144	1.031	−1.421	1.001	0.380
0x000C	-	-	0.988	−0.352	0.998	−1.072	1.004	−2.376	0.991	0.288
	-	-	0.994	−0.173	1.005	−0.854	1.027	−0.704	0.998	0.479

^1^ Confidence intervals calculated from the initial linear regression for the BMP390 pressure measurement were not valid after applying the 1-point calibration.

**Table 8:** Goodness of fit statistics, coefficient of determination (R^2^), and root mean square error (RMSE) for calibration data.

Serial Number	BMP390				RTD		SHT40			
	Barometric Pressure (Pa)		Temperature (°C)		Temperature (°C)		Relative Humidity (%)		Temperature (°C)	
	R^2 α^	RMSE ^β^	R^2^	RMSE	R^2^	RMSE	R^2^	RMSE	R^2^	RMSE
0x0005	-	238.350	0.9999	0.074	0.9999	0.080	0.9986	0.622	0.9999	0.076
0x0006	-	304.462	0.9999	0.085	0.9999	0.088	0.9984	0.653	0.9999	0.086
0x0007	-	303.454	0.9998	0.107	0.9998	0.115	0.9980	0.742	0.9998	0.111
0x0008	-	308.586	0.9998	0.119	0.9997	0.143	0.9977	0.787	0.9998	0.129
0x0009	-	293.523	1.0000	0.054	0.9999	0.065	0.9986	0.624	1.0000	0.058
0x000A	-	279.854	1.0000	0.059	0.9999	0.069	0.9986	0.625	0.9999	0.064
0x000B	-	259.870	0.9999	0.077	0.9999	0.087	0.9983	0.674	0.9999	0.083
0x000C	-	264.826	0.9999	0.098	0.9998	0.117	0.9979	0.750	0.9998	0.106

^α^ Coefficients of determination calculated from the initial linear regression for the BMP390 pressure measurement were not valid after applying the 1-point calibration. ^β^ Root mean square error calculated for the BMP390 pressure measurements was calculated directly from the PTH probe and reference instrument instead of using the linear regression model.

**Table 9:** Tukey-Kramer grouping for least-squares means (LSM) of PTH probe sensors during calibration run. Within a column, LSMs with the same letter are not statistically significant.

Serial Number	BMP390					RTD		SHT40					
	Barometric Pressure (Pa)		Temperature (°C)			Temperature (°C)		Relative Humidity (%)			Temperature (°C)		
	LSM	Group	LSM	Group		LSM	Group	LSM ^1^	Group		LSM	Group	
0x0005	98,516.6411	G	29.3928		E	29.7934	C	4.2124		D	28.7023	C	B
0x0006	98,582.9873	B	29.5137	B	A	28.9190	F	4.2105	E	F	28.6909	C	B
0x0007	98,581.8877	B	29.4979	B		30.3079	A	4.2119	E	D	28.7665		A
0x0008	98,587.2903	A	29.5314		A	29.9159	B	4.2113	E	D	28.7964		A
0x0009	98,571.6201	C	29.3790		E	29.4872	D	4.2183		A	28.6499		D
0x000A	98,558.5613	D	29.4231		D	28.9725	E	4.2167		B	28.6756	C	D
0x000B	98,538.1433	F	29.4585		C	28.6805	G	4.2143		C	28.7158		B
0x000C	98,543.1564	E	29.5089	B	A	29.9090	B	4.2093		F	28.7702		A

^1^ Logarithmically transformed least-squares means.

**Table 10:** Regression model coefficients for calibrated PTH probes.

Serial Number	BMP390				RTD		SHT40			
	Barometric Pressure (Pa)		Temperature (°C)		Temperature (°C)		Relative Humidity (%)		Temperature (°C)	
	Slope ^1^	Offset	Slope	Offset	Slope	Offset	Slope	Offset	Slope	Offset
0x0005	1.0000	−3.5055	1.0014	−0.0534	1.0010	−0.0372	0.9949	0.3863	1.0014	−0.0497
0x0006	1.0000	−2.6123	1.0026	−0.0855	1.0014	−0.0544	0.9947	0.4013	1.0023	−0.0736
0x0007	1.0000	−2.3942	1.0037	−0.1166	1.0035	−0.1086	0.9953	0.3274	1.0038	−0.1158
0x0008	1.0000	−4.4952	1.0044	−0.1435	1.0045	−0.1380	0.9953	0.3059	1.0046	−0.1400
0x0009	1.0000	−3.7746	1.0001	−0.0149	0.9993	0.0037	0.9941	0.4681	1.0000	−0.0090
0x000A	1.0000	−3.7360	1.0008	−0.0338	1.0001	−0.0162	0.9942	0.4568	1.0006	−0.0285
0x000B	1.0000	−3.7677	1.0022	−0.0778	1.0016	−0.0550	0.9942	0.4334	1.0021	−0.0717
0x000C	1.0000	−1.6503	1.0034	−0.1087	1.0032	−0.1018	0.9944	0.3814	1.0035	−0.1078

^1^ Manually set to 1.000 for 1-point calibration.

**Table 11:** Lower and upper bounds of the 95% confidence interval for calibration coefficients.

Serial Number	BMP390				RTD		SHT40			
	Barometric Pressure (Pa)		Temperature (°C)		Temperature (°C)		Relative Humidity (%)		Temperature (°C)	
	Slope ^1^	Offset ^1^	Slope	Offset	Slope	Offset	Slope	Offset	Slope	Offset
0x0005	-	-	0.9988	−0.1281	0.9980	−0.1231	0.9826	−0.4630	0.9986	−0.1295
	-	-	1.0040	0.0214	1.0039	0.0487	1.0073	1.2356	1.0041	0.0300
0x0006	-	-	0.9997	−0.1692	0.9982	−0.1455	0.9818	−0.4866	0.9992	−0.1606
	-	-	1.0055	−0.0017	1.0046	0.0367	1.0076	1.2893	1.0053	0.0134
0x0007	-	-	1.0003	−0.2144	0.9996	−0.2193	0.9813	−0.6420	1.0002	−0.2202
	-	-	1.0071	−0.0189	1.0073	0.0021	1.0094	1.2968	1.0075	−0.0114
0x0008	-	-	1.0008	−0.2478	0.9999	−0.2694	0.9807	−0.7000	1.0005	−0.2564
	-	-	1.0080	−0.0393	1.0090	−0.0066	1.0099	1.3118	1.0086	−0.0237
0x0009	-	-	0.9979	−0.0785	0.9966	−0.0730	0.9819	−0.3702	0.9976	−0.0778
	-	-	1.0023	0.0487	1.0019	0.0804	1.0063	1.3065	1.0023	0.0598
0x000A	-	-	0.9985	−0.0994	0.9974	−0.0963	0.9817	−0.4088	0.9981	−0.1011
	-	-	1.0031	0.0318	1.0029	0.0640	1.0068	1.3225	1.0032	0.0441
0x000B	-	-	0.9996	−0.1550	0.9985	−0.1448	0.9805	−0.5083	0.9992	−0.1578
	-	-	1.0049	−0.0007	1.0047	0.0349	1.0078	1.3751	1.0051	0.0144
0x000C	-	-	1.0002	−0.1996	0.9993	−0.2157	0.9795	−0.6438	0.9999	−0.2102
	-	-	1.0065	−0.0177	1.0072	0.0120	1.0093	1.4067	1.0070	−0.0053

^1^ Confidence intervals calculated from the initial linear regression for the BMP390 pressure measurement were not valid after applying the 1-point calibration.

**Table 12:** Goodness of fit statistics, coefficient of determination (R^2^), and root mean square error (RMSE) for validatsion data.

Serial Number	BMP390				RTD		SHT40			
	Barometric Pressure (Pa)		Temperature (°C)		Temperature (°C)		Relative Humidity (%)		Temperature (°C)	
	R^2 α^	RMSE ^β^	R^2^	RMSE	R^2^	RMSE	R^2^	RMSE	R^2^	RMSE
0x0005	-	24.732	0.9999	0.072	0.9999	0.083	0.9981	0.645	0.9999	0.077
0x0006	-	25.570	0.9999	0.080	0.9999	0.088	0.9980	0.675	0.9999	0.084
0x0007	-	25.519	0.9999	0.094	0.9998	0.106	0.9976	0.736	0.9998	0.100
0x0008	-	24.438	0.9998	0.100	0.9997	0.126	0.9974	0.763	0.9998	0.112
0x0009	-	30.141	0.9999	0.061	0.9999	0.074	0.9982	0.638	0.9999	0.066
0x000A	-	22.536	0.9999	0.063	0.9999	0.077	0.9981	0.658	0.9999	0.070
0x000B	-	26.302	0.9999	0.074	0.9999	0.086	0.9977	0.716	0.9999	0.083
0x000C	-	25.947	0.9999	0.087	0.9998	0.109	0.9973	0.779	0.9998	0.098

^α^ Coefficients of determination calculated from the initial linear regression for the BMP390 pressure measurement were not valid after applying the 1-point calibration. ^β^ Root mean square error calculated for the BMP390 pressure measurements was calculated directly from the PTH probe and reference instrument instead of using the linear regression model.

**Table 13:** Tukey-Kramer grouping for least-squares means (LSM) of PTH probe sensors during validation run. Within a column, LSMs with the same letter are not statistically significant.

Serial Number	BMP390					RTD		SHT40					
	Barometric Pressure (Pa)			Temperature (°C)		Temperature (°C)		Relative Humidity (%)				Temperature (°C)	
	LSM	Group		LSM	Group	LSM	Group	LSM ^1^	Group			LSM	Group
0x0005	97,804.0231	B	A	27.7549	A	27.7505	A	4.1978	B	A	C	27.7516	A
0x0006	97,803.1300	B	A	27.7530	A	27.7557	A	4.1978	B	A	C	27.7507	A
0x0007	97,802.9118	B	A	27.7542	A	27.7522	A	4.1984	B	A		27.7494	A
0x0008	97,805.0129		A	27.7621	A	27.7538	A	4.1988		A		27.7538	A
0x0009	97,804.2922	B	A	27.7524	A	27.7561	A	4.1974			C	27.7503	A
0x000A	97,804.2536	B	A	27.7521	A	27.7526	A	4.1974	B		C	27.7505	A
0x000B	97,804.2854	B	A	27.7556	A	27.7503	A	4.1979	B	A	C	27.7522	A
0x000C	97,802.1679	B		27.7551	A	27.7524	A	4.1985		A		27.7516	A

^1^ Logarithmically transformed least-squares means.

## Data Availability

The raw data presented in this study are available on request from the corresponding author.
